# Cases of Oral Surgery

**Published:** 1889-06

**Authors:** Nelson C. Dobson

**Affiliations:** Surgeon to the Bristol General Hospital, &c.


### CASES OF ORAL SURGERY. By NELSON C. DOBSON, F.R.C.S., Surgeon to the Bristol General Hospital, &c.

I HAVE now under my care a series of cases illustrating several phases of the surgery of the mouth, of which I select the five following examples :

Case I.—Excision of superior maxilla for sarcomatous disease.

A woman, aged 54, first came under my care in 1885. She then complained of severe neuralgic pains in the left side of her face ; but there was no sufficient reason to account for this. The neuralgia was persistent, and did not yield to medicines. I went away for my summer holiday; and on my return, noticed that the upper jaw, above the alveolus, was thinned; and there was some obstruction to the left nostril. There was now no doubt that there was a rapidly-growing tumour in her antrum. Accordingly, I took her into the hospital, and removed her superior maxilla on Sept. 10th. I did a preliminary laryngotomy, which materially facilitated the operative proceedings, and allowed of the uninterrupted administration of chloroform. The patient made a good recovery, and left the hospital in a few weeks. The growth was a sarcomatous one, but I have no distinct record of the microscopic appearance. She remained well for some time; but early in 1887 she showed signs of recurrence, as seen in the mouth, but more especially in the temporal and zygomatic region. I suggested, but did not press, further operative proceedings. From this time onwards she suffered much, and there was considerable swelling and some suppuration about orbit and temporal region.

The eyeball was becoming prominent, and her local condition was such that I thought no further active interference wise or possible. However, after considerable suppuration, the mass in the mouth seemed to subside and the external swelling to diminish, and in January, 1889, I removed the projecting and spoiled eyeball, and the patient is now fairly well. It seemed as if the sarcomatous growth had suppurated out, and the disorder appears to be declining. The points of interest appear to be: the neuralgic and altogether subjective symptoms in the early part of the malady; the recurrence and sloughing, which seem to have led to more or less of a cure; and the question of preliminary laryngotomy in this and similar cases.

Case II. was an instance of epithelioma beginning in the alveolus and soft parts of the hard palate. A lady, æt. 74, was sent to me by one of my medical friends with an extensive epithelioma of the alveolus and soft parts of the palate on the left side: the disorder had reached an advanced stage when I first saw this patient in April, 1888, and there was, in addition to a well-marked epithelioma of the palate, very considerable enlargement of the cervical glands. The patient suffered much pain, so that taking food was a matter of considerable difficulty. She did not appear to appreciate the gravity of her condition; and though I could not promise much respite from her present suffering, she consented for me to do what I could for her under the circumstances. Therefore, on May 1st, 1888, I dissected off and scraped, and gouged away all the diseased structures as far as was possible. This was a work of some difficulty, and there was pretty free bleeding. I did not attempt to remove the glands, as I explained to the patient that it was not possible to relieve her for more than a short time of the extreme suffering she was having from the growth in the mouth. I saw this patient again in November last: there was then some recurrence in the mouth, and the cervical glands were distinctly larger; but she had been free from pain since May last. Of course there was only the prospect of steady increase in the disease. This case was an illustration of palliation of the disorder for a limited period.

Case III.—A woman (E. A.), aged 60, applied at the hospital with a sore over her gum of the lower jaw, on the right side, extending into the floorof the mouth: there was a projecting tooth in the upper jaw, which appeared to irritate the sore. I had no doubt the condition was one of epithelioma; but as it appeared to spring from the alveolus and to be fairly localised, I thought I could remove it from within the mouth by dissection, scraping, and gouging of bone. This I did on November 8th, but the disorder proved more extensive than I imagined. Although I was fairly satisfied with this proceeding, yet I was doubtful whether my removal had been sufficiently extensive: there were, however, soon signs that a more extended operation would be necessary; and on December 13th, 1888, I removed the right half of the horizontal ramus of the lower jaw, together with enlarged submaxillary glands and all the soft parts constituting the floor of the mouth on that side. To do this I cut down the median line of the lip and reflected the skin along the lower border of the jaw as far as the angle; a drainage-tube was brought out through the incision, and the wound healed perfectly. The soft parts contracted, and the difficulty in moving the jaw and the apparent deformity was wonderfully little. I fear, however, now (March 13) there is a tendency for the cut on the horizontal ramus to show signs of recurrence, and I expect I shall remove the remaining part of the horizontal ramus. I have on a former occasion removed the entire half of the lower jaw, including condyle, and I have also removed simultaneously the greater part of both superior maxillæ; but I have never, up to now, removed the whole of the horizontal ramus of the lower jaw. The disorder in this case appears to have commenced in the alveolus, and to have extended to the bone itself; for it is the bone which is most actively taking on fresh action.

Since this paper was written, I have removed the whole of the horizontal ramus which was left after the first operation.

Case IV. is a case of malignant tumour of the tongue in a man of 68, and on whom I operated on January 31st, 1889. His family history was good, and there was no history of syphilis. He had only been complaining of discomfort in his mouth for about two months before I saw him; though I have no doubt the tumour has been growing longer than this time. When I first saw him on January 26th, 1889, the left side of the tongue, to quite close on to the epiglottis, was occupied by a firmish tumour the size of a walnut: the outline of the swelling was smooth, and the mucous membrane unbroken, except a small patch on the inner margin. The appearance of this small sore was that of an ordinary epithelioma; the swelling was well limited to the left half of the tongue. There was no glandular enlargement. I determined to remove only half of the tongue, which I accordingly did on January 31st. The patient being under chloroform and the mouth opened by a gag placed on the right side, I transfixed the tip of the tongue by a stout ligature on each side, and then, holding the tongue upwards, I cut along the floor of the mouth quite close to the jaw, dividing freely the mucous membrane as far back as the anterior palatine arch; I also cut across the anterior pillar of the fauces. Next I cut freely down the exact middle line of the dorsum, dividing freely the mucous membrane in this situation, and completely dividing all the structures of the tip of the tongue: then, taking the ligatures which were passed through the tip, one in each hand, and drawing them towards their respective sides of the mouth, I quickly and easily, with my index-fingers, divided the tongue into two halves. I now took the ligature attached to the half I intended to remove and drew it forcibly upwards and forwards, dividing with scissors any tissues which prevented this half of the tongue being freely drawn out: when I judged I had sufficiently freed the tongue, I thrust a strong curved needle, on a handle, beyond the limits of the disease, and quickly cut off the tongue with a small wire ecraseur. Just before the final separation of the tongue by the wire there appeared to be only vessels in the loop, and I therefore passed a catgut ligature around this and divided it. During the operation, which is the one advocated by Mr. Morrant Baker, there was very little hæmorrhage or difficulty of any kind. With the exception of Whitehead's method, which I have never practised, and of which therefore I cannot speak, this seems to me the easiest, simplest, and safest operation for removal of one half or the whole of the tongue. I have several times operated on the tongue, but this was certainly the easiest operation I have ever done of its kind. Unfortunately, it is not suitable to cases where the floor of the mouth is much involved. In such cases I have found Symes' operation to be the best. The tumour presents on section a wellmarked and distinct outline, and this case especially looks more like a sarcoma than an epithelioma, though sarcoma of tongue is exceedingly rare.

Case V. is a case of epulis growing in the mouth of a man aged 19, and is chiefly remarkable from its size and for the fact that an individual could in these days allow the growth to assume such proportions. The tumour, as far as could be ascertained prior to operation, sprang from the alveolus of the right side of the lower jaw. He states that it has been growing seven years and that once an attempt was made to remove it, but it was not then thoroughly removed. When I saw the patient in February last, the tumour filled the right side of his mouth, pressing the tongue backwards; it also projected to near the roof of the mouth, and caused a considerable bulging of the lower lip. I could not quite define its limits, but felt that it was attached to the alveolus, and I was prepared to remove a portion of the whole thickness of the jaw: however, I succeeded in chiselling off the alveolus with the tumour attached; the hæmorrhage was pretty free from the bone, which I stopped by pressure and thermo-cautery. The tumour, which is as large as a fair-sized apple, is mostly fibrous in structure, with bony fragments scattered through it. It is the largest tumour of its kind I have ever seen.

